# The role of oxygen-increased respirator in humans ascending to high altitude

**DOI:** 10.1186/1475-925X-11-49

**Published:** 2012-08-16

**Authors:** Guanghao Shen, Kangning Xie, Yili Yan, Da Jing, Chi Tang, Xiaoming Wu, Juan Liu, Tao Sun, Jianbao Zhang, Erping Luo

**Affiliations:** 1 Key Laboratory of Biomedical Information Engineering of Ministry of Education, School of Life Science and Technology, Xi’an Jiaotong University, Xi’an, Shaanxi, 710049, P. R China; 2 School of Biomedical Engineering, Fourth Military Medical University, Xi’an, Shaanxi, 710032, P. R China

**Keywords:** Oxygen-increased respirator, Heart rate, Free radical, Acute mountain sickness

## Abstract

**Background:**

Acute mountain sickness (AMS) is common for people who live in low altitude areas ascending to the high altitude. Many instruments have been developed to treat mild cases of AMS. However, long-lasting and portable anti-hypoxia equipment for individual is not yet available.

**Methods:**

Oxygen-increased respirator (OIR) has been designed to reduce the risk of acute mountain sickness in acute exposure to low air pressure. It can increase the density of oxygen by increasing total atmospheric pressure in a mask. Male subjects were screened, and eighty-eight were qualified to perform the experiments. The subjects were divided into 5 groups and were involved in some of the tests at 4 different altitudes (Group 1, 2: 3700 m; Group 3,4,5: 4000 m, 4700 m, 5380 m) with and without OIR. These tests include heart rate, saturation of peripheral oxygen (SpO_2_), malondialdehyde (MDA), superoxide dismutase (SOD), blood lactate (BLA) and PWC (physical work capacity) -170.

**Results:**

The results showed that higher SpO_2_, lower heart rate (except during exercise) and better recovery of heart rate were observed from all the subjects ’with OIR’ compared with ’without OIR’ (P<0.05). Moreover, compared with ’without OIR’, subjects ’with OIR’ in Group 1 had lower concentrations of MDA and BLA, and a higher concentration of SOD (P<0.05), while subjects ’with OIR’ in Group 2 showed better physical capacity (measured by the PWC-170) (P<0.05). The additional experiment conducted in a hypobaric chamber (simulating 4,000 m) showed that the partial pressure of oxygen in blood and arterial oxygen saturation were higher ’with OIR’ than ’without OIR’ (P<0.05).

**Conclusions:**

We suggested that OIR may play a useful role in protecting people ascending to high altitude before acclimatization.

## Background

Plateaus, known variously as tablelands, or high altitude, are areas elevated thousands of meters above sea level. With the recent opening of Qinghai-Tibet railway in China, more and more people who live in low altitude areas enter Qinghai-Tibetan high plateau for work, science investigation or tour. Many of them experience acute mountain sickness (AMS), suffering from hypoxia due to the low partial pressure of oxygen at increasing altitudes. The most common symptoms of AMS, including headache, poor appetite, nausea, fatigue, dizziness and insomnia, usually appear within the first three days of arrival at high altitude [1]. Moreover, people from low altitudes may experience a decrease in exercise performance when ascending to high altitude. The higher the altitude is, the more critical the symptoms may become. This problem may seriously influence the physical and mental state and work efficacy for travelers and workers.

To reduce the risk of AMS, many instruments have been developed, including bottled oxygen [2], portable hyperbaric chamber [3-5] and oxygen enrichment room [6]. The supplemental oxygen provided by these instruments raises the oxygen concentration and reduces the equivalent altitude [7,8], which can treat mild cases of AMS. However, long-lasting and portable anti-hypoxia equipment for individual is not yet available.

In this study, we performed a prospective, self-controlled study to determine the effects of oxygen-increased respirator (OIR, custom-made instrument) on changes in (1) heart rate and saturation of peripheral oxygen (SpO_2_) [9] in four different altitudes; (2) malondialdehyde (MDA), superoxide dismutase (SOD), blood lactate (BLA) [10] at 3,700 m; and (3) PWC (physical work capacity)-170 test [11] at 3,700 m; (4) arterial blood gas analysis at simulated altitude of 4000 m. During each session, subjects performed the experiment with or without OIR.

## Materials and methods

### Subjects

The subjects, coming from a road construction company, were going to high altitude to build roads. After signing informed consent forms, subjects completed a structured questionnaire covering anthropometric variables, lifestyle questions, and medical history. Meanwhile, our research faculties followed them to the high altitude from Xi’an city (430 m) by vehicles in five days. The field working bases with hotel facilities were distributed into the four different altitudes (3700 m, 4000 m, 4700 m, and 5380 m). Accordingly, our research faculties were organized into four teams to carry out the experiment in specific altitude (Team 1–4).

At each altitude, the subjects were screened. Some were excluded when not suitable for testing. Exclusion criteria included cardiac illness, diuretic use, chronic medical conditions, or previous experience of AMS. All of the 88 qualified subjects were young males, of similar height (from 169 to 172 cm) and weight (from 68 to 72 kg), ranging in age from 20 to 24 years. They were divided into 5 groups, 34 at 3700 m (17 in each of Group 1 and 2), 18 at 4000 m (Group 3), 19 at 4700 m (Group 4), 17 at 5380 m (Group 5).

Additional 8 young men (22–24 years old) in Xi’an city were selected for the blood gas tests in hypobaric chamber.

The study was approved by the local institutional ethical review boards of all the participating institutions.

#### Instrument

**Figure 1:**
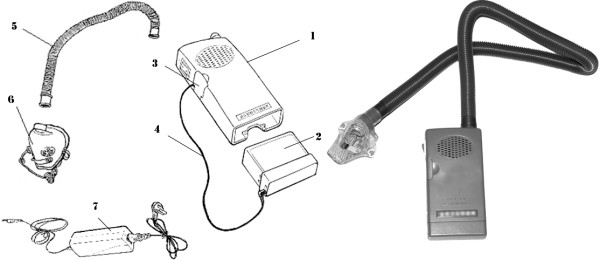
** Schematic diagram of OIR.1) **machine body** 2)** battery **3)** wire chamber** 4)** extending power wire** 5) **air tube **6) **mask **7) **charger.

To satisfy needs in high altitude, the OIR was invented (Figure 1, Chinese patent No. 200610104868.1, 200630090257.7, 200403262498.0). The total weight is 370 g, and the dimensions are 152 × 79 × 34 mm. As a simple, portable, durable, and convenient device, the OIR increases atmospheric pressure and therefore oxygen partial pressure in a nasal mask by means of a centrifugal fan with a rotational speed of 10000 rpm. Magnetic suspension bearing was adopted to reduce noise and improve power consumption ratio. Conveniently, it uses internal rechargeable Li-ion batteries (11.1 V, 1800 mAh) which, under extreme cold environment, can be displaced under the user’s clothes with an extended wire connecting to the machine body to be kept warm and working.

The instrument does not change the percentage of oxygen (approximately 21%) in the compressed atmosphere. There are 12 small holes (2.5 mm in diameters) in the mask allowing air exchanges. The output pressure in the mask, as measured using a U-tube manometer, is 3 mmHg above the local atmospheric pressure; flow rate of atmosphere is approximately 50 L/min. The edge of the nasal mask is made of polysiloxanes, which can fit different faces and comfort the wearers.

#### Experiment design

**Figure 2:**
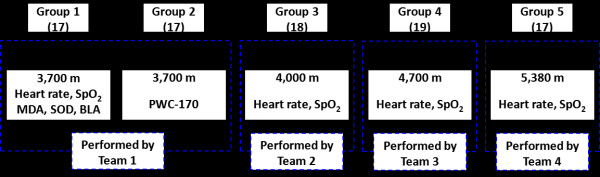
The details of the experimental design.

The details of the experimental design are shown in Figure 2. The base at 3700 m has better facilities and two Groups (Group 1 and 2) were assigned with more tests performed than other groups. In Group 1, heart rate, SpO_2_, MDA, SOD and BLA were tested. On day one, subjects wore the OIR. Team 1 recorded the subjects’ heart rates and SpO_2_ at rest by a Multi-parameter patient monitor (IntelliVue MP70, Philips, Eindhoven, The Netherlands). Next, all subjects exercised using Harvard Step Test designed to induce fatigue [12]. The height of each step is 40 cm; subjects went up and down at a rate of 20 steps per minute for 3 minutes. The heart rate and SpO_2_ were recorded at the end of exercise, and the recovery of heart rate was tracked during a rest period of 5 minutes. Immediately after the excise, the vein blood was drawn from the antecubital fossa to assess the concentrations of MDA [13], SOD [14], and BLA with three diagnostic kits (Jiancheng Bioengineering Institute, Nanjing, China) [15]. The experimental protocol was repeated without OIR on day two after a rest period of 24 hours.

PWC-170 [16] were tested from another 17 subjects in Group 2. They performed consecutive workloads on a cycle ergometer (EGM-II, Yueyang Electronic Ins., Co., Hunan, China) with OIR. Subjects were asked to keep the tachometer at around 60 rpm. Loaded power of the ergometer was initially set to 50 W, then increased by a step of 50 W per 3 minutes until reaching 200 W. Heart rate was monitored until a steady read-out was achieved. Each steady-state heart rate and workload were graphed, with the line of best fit for the three points extrapolated to estimate the power out that would elicit a heart rate of 170 beats per minute [16,17]. The experimental protocol was repeated without OIR after a rest of 24 hours.

At higher altitude (4,000 m, 4,700 m and 5,380 m for Group 3, 4, 5 respectively), only heart rate and SpO_2_ were measured from subjects exercising Harvard Step Test with the same type of patient monitor. Subjects performed the experiments first with OIR and then repeated without OIR after a rest of 24 hours.

An additional experiment was conducted to verify the exact arterial partial pressure of oxygen in blood (PaO_2_) and arterial oxygen saturation (SaO_2_) by measuring arterial blood gas with a portable clinical analyzer (i-STAT 200, Abbott Point of Care Inc., USA). Eight young men were exposed in the hypobaric chamber simulating high altitude of 4000 m. Blood samples were collected from each subject after 2 hours exposure (without OIR). Afterwards, OIRs were used for 15 minutes and blood samples were collected (with OIR).

#### Statistical analysis

All of the data are shown as means ± S.D. We used SPSS 13.0 software (SPSS Inc., Chicago, IL, USA) to perform Shapiro-Wilk normality test and paired *t*-test. P<0.05 was considered statistically significant.

## Results

**Figure 3:**
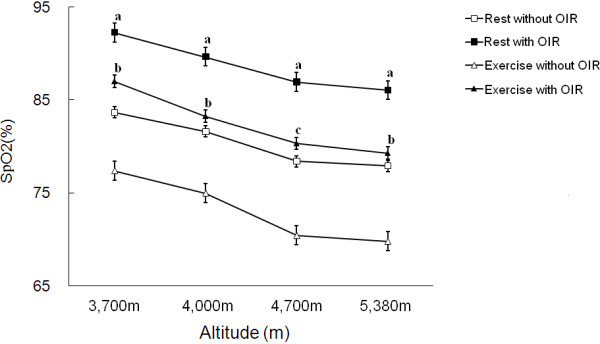
** Effects of OIR on SaO2 at high altitudes of 3,700 m, 4,000 m, 4,700 m, and 5,380 m.** P values were estimated using paired Student’s *t*-test. aP < 0.05, as compared with ’Rest without OIR’. bP < 0.05 and cP < 0.01, as compared with ’Motion without OIR’.

**Figure 4:**
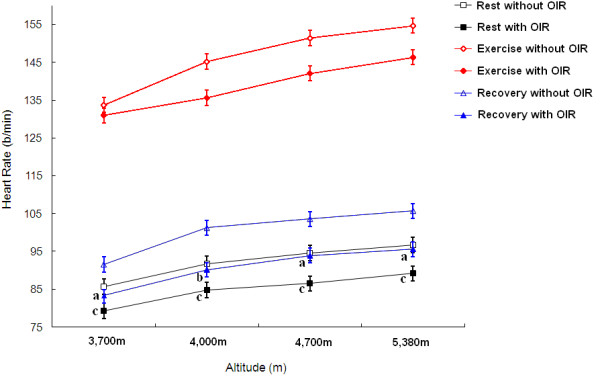
** Effects of OIR on heart rate at high altitudes of 3,700 m, 4,000 m, 4,700 m, and 5,380 m.** a P<0.05 and b P<0.01, as compared with ’Recovery without OIR’. c P<0.05, as compared with ’Rest without OIR’.

Both in the situations of rest and of exercise, the results showed that the subjects with OIR had a higher SpO_2_ than the same subjects without OIR (P<0.01 in exercise at 4700 m, P<0.05 in other circumstance, Figure 3). Compared with the ’without OIR’ group, subjects with OIR had a lower heart rate at rest and better recovery of heart rate after exercise (P<0.01 in recovery at 4000 m, P<0.05 in other circumstance, Figure 4); however, there was no significant difference in heart rate during exercise between the two conditions. Compared with ’without OIR’, subjects ’with OIR’ in Group 1 had lower concentrations of MDA and BLA, and a higher concentration of SOD (P<0.05, Table 1). Based on the performances of PWC-170, subjects with OIR presented higher physical capacity. The power output of the subjects with OIR was better than that when without OIR (P<0.05, Table 2).

**Table 1:** Changes in MDA, SOD, and BLA at 3,700 m (Mean ± SD, N = 17)

	**MDA (μmol/L)**	**BLA (mmol/L)**	**SOD (U/L)**
Without OIR	4.51 ±0.22	6.37 ±0.53	95.35 ±13.59
With OIR	4.33 ±0.25*	5.42 ±0.56*	112.62 ±12.93*

*P<0.05. ’Without OIR’ vs. ’With OIR’. P values were estimated using paired Student’s *t*-test. MDA: Malonodialdehyde, SOD: Superoxide dismutase, BLA: Blood lactate.

**Table 2:** Increased output Power (watt) by using OIR in the PWC-170 test (Mean ± SD, N = 17)

**Heart rate range(BPM)**	**115 -130**	**130 -145**	**170**^**e**^
Without OIR	100.9 ± 12.1	158.2 ± 16.2	196.3 ± 15.5
With OIR	119.6 ± 9.1*	182.1 ± 10.5*	223.5 ± 11.3*

170^e^:These data were estimated by three consecutive workloads.

*P<0.05. ’Without OIR’ vs. ’With OIR’. P values were estimated using paired Student’s *t*-test.

Comparing the results from Group 3, 4 and 5, we found the following: First, the SpO_2_ with OIR was higher than that without OIR both at rest and during exercise (Figure 3). Second, the heart rate with OIR was lower than that without OIR at rest and during the 5-minute recovery period (Figure 4).

In the simulation experiment, we found that PaO_2_ and SaO_2_ at altitude of 4000 m simulated in the hypobaric chamber were higher for the ’with OIR’ group than for ’without OIR’ group (P<0.05, Table 3).

**Table 3:** Arterial blood gas and oxygen saturation in altitude of 4000 m simulated in the hypobaric chamber (Mean ± SD, n = 8).

	**pH**	**PaCO**_**2**_**(mmHg)**	**PaO**_**2**_**(mmHg)**	**SaO**_**2**_**(%)**
Without OIR	7.45 ± 0.03	35.2 ± 2.9	55.0 ± 3.5	68.3 ± 5.9
With OIR	7.49 ± 0.05	31.3 ± 4.2	63.2 ± 3.3*	80.3 ± 9.4*

PaCO_2_: the partial pressure of carbon dioxide; PaO_2_: the partial pressure of oxygen; SaO_2_: arterial oxygen saturation. *P<0.05. ’Without OIR’ vs. ’With OIR’. P values were estimated using paired Student’s *t*-test.

## Discussion

The OIR was developed with the aim of protecting unacclimatized people who must ascend to high altitude. The edge of the nasal mask is made of polysiloxanes, which can fit different faces and make wearers feel comfortable. In our study, we tested the effects of OIR at high plateau in subjects (young men aged 20–24 years old) at conditions of rest or exercise. Subjects with OIR had lower heart rates (except during exercise), lower concentrations of MDA and BLA, higher levels of SpO_2_ and concentration of SOD, and better physical capacity than the same subjects without OIR. Some researchers reported similar findings in unsystematic and single-trial studies using smaller samples and/or different methods [18,19]. However, we have to point out that the subjects in this study are healthy male subjects and results may differ for other subjects.

The percentage of oxygen in air is constant, remaining at 21% in high altitude; however, atmospheric pressure is inversely related to altitude, making the amount of oxygen available much less in high altitude. The effect of ascending high altitude without acclimatization is a pathological condition that is caused by acute exposure to low atmospheric pressure [1]. Therefore, the key factor of AMS was the partial pressure of oxygen, the product of overall pressure and fractional concentration of oxygen.

The oxyhemoglobin dissociation curve describes the non-linear relation between the partial pressure of oxygen in the blood and the oxygen saturation. The sigmoid shape of the curve indicates that small increase in partial pressure of oxygen can lead to high increase of SaO_2_. In our study, although the OIR just slightly increases the oxygen partial pressure of inspiratory gas in the airway (the output pressure of OIR is measured at about 3 mmHg above the local atmospheric pressure), it significantly increases the SpO_2_ (about 12.8%). Caution must be taken that the added partial pressure of oxygen by using the OIR is exerted in the airway and not necessarily equally transferred into the blood. In fact, we did an additional experiment verifying the exact arterial partial pressure of oxygen in blood by measuring arterial blood gas. The additional experiment on blood gas confirmed the SaO_2_ in blood is high with OIR than without OIR.

SpO_2_ is the indirect measure (by a pulse oximetry at fingertip) of the ratio of oxyhemoglobin to the total concentration of hemoglobin present in the blood. Heart rate has a close relationship to oxygen consumption, and heart rate increases following ascent to high altitude. The recovery of heart rate, measured at a fixed (or reference) period after ceasing activity, is correlated to some factors which affect cardiovascular functions [20]. In ’with OIR’ condition, OIR significantly enhanced SpO_2_ and heart rate recovery after exercise and reduced heart rate during rest at all altitudes. In particular, SpO_2_ at 4,700 m and heart rate recovery at 4,000 m were highly significantly different between with and without OIR. A higher level of SpO_2_ should protect cardiopulmonary function during acute exposure to low atmospheric pressure, and may also play an important role in reducing heart overload.

Lassitude is one of the symptoms of altitude sickness [21]. Erythrocyte membranes are perhaps the most exposed to peroxidative damage by free radicals [22]. In low atmospheric pressure conditions, the imbalance between oxidation and antioxidation reactions results in a higher concentration of MDA, which further impairs cell function [23] and damages cellular membrane components [24]. BLA is used clinically as an indicator of circulatory impairment as well as the overall state of oxygenation [25]. Antioxidant defenses in the red cell can temper the negative effect of free radicals and related reactions and keep them in check. SOD forms a substantial defense network against oxidative stress imposed by physical activity [3]). Subjects in Group 1 with OIR had lower concentrations of MDA and BLA, and a higher concentration of SOD (P<0.05) at 3,700 m, compared with when without OIR. This suggests that OIR works by reducing lipid peroxidation and cell membrane damage, thereby increase the body’s ability to fight anoxia and fatigue.

Flueck et al. [11] reported that the physical capacity for work can be tested in young males aged 17–24 by means of the PWC-170 test. The PWC-170 has also been shown to be a valid tool at high altitude [26]. The OIR increased air flow capacity by the rotating fan and accumulated the volume of oxygen. The higher total air pressure and oxygen saturation in subjects using OIR sustained normal physical alertness in high altitude, as assessed by their performance in the PWC-170 test.

OIR has two known limitations. First, OIR has not been proved to treat AMS, despite the data showing better performance. Second, OIR may cause irritation of the respiratory tract when air temperature is low. One limitation of the experimental design is that all the subjects performed experiments with OIR first. In future work, an independent experiment concerning the sequence of with and without OIR should be designed to reveal its effects on the results.

## Conclusions

The OIR not only increased the level of SpO_2_, concentration of SOD, and physical capacity but also reduced the heart rate (except during exercise) and concentrations of MDA and BLA, which play important protective roles at low atmospheric pressures and reduce the risk of AMS. We suggested that OIR may play a useful role in protecting people needing to ascend to high altitude before acclimatization.

## Competing interests

The authors declare that they have no competing interests.

## Authors’ contributions

EL: conceived and designed the study, was involved in funding application, carried out data acquisition, analysis and interpretation, drafted and revised the manuscript. JZ: participated in the coordination of the research group, involved in the acquisition of funding, participated in the writing and revision of the manuscript. GS: participated in the experimental tests, drafted and revised the manuscript. KX: participated in the experimental tests, drafted and revised the manuscript. YY: participated in the experimental tests, carried out data acquisition. CT: carried out data acquisition. XW, JL, TS: carried out data analysis. All authors read and approved the final manuscript.
